# A Pilot Randomised Double-Blind Study of the Tolerability and efficacy of repetitive Transcranial Magnetic Stimulation on Persistent Post-Concussion Syndrome

**DOI:** 10.1038/s41598-019-41923-6

**Published:** 2019-04-02

**Authors:** Zahra Moussavi, Abdelbaset Suleiman, Grant Rutherford, Omid Ranjbar Pouya, Zeinab Dastgheib, Weijia Zhang, Jennifer Salter, Xikui Wang, Behzad Mansouri, Brian Lithgow

**Affiliations:** 1 Biomedical Engineering, University of Manitoba, Winnipeg, Canada; 2 Riverview Health Centre, Winnipeg, Canada; 3 Statistics Department, University of Manitoba, Winnipeg, Canada; 4 Neurology Department, University of Manitoba, Winnipeg, Canada; 5 Monash Alfred Psychiatry Research Center, Melbourne, Australia

## Abstract

This study investigates the effect of Repetitive Transcranial Magnetic Stimulation (rTMS) on persistent post-concussion syndrome (PCS). The study design was a randomized (coin toss), placebo controlled, and double-blind study. Thirty-seven participants with PCS were assessed for eligibility; 22 were randomised and 18 completed the study requirements. Half the participants with PCS were given an Active rTMS intervention and the other half given Sham rTMS over 3 weeks. Follow ups were at the end of treatment and at 30 and 60 days. The primary outcome measure was the Rivermead Post-Concussion Symptoms Questionnaire (RPQ3 & RPQ13). The results indicate participants with more recent injuries (<12 month), who received Active rTMS, showed significant improvements compared to those of: 1) the same subgroup who received Sham, and 2) those with a longer duration of injury (>14 months) who received Active rTMS. This improvement predominantly manifested in RPQ13 in the follow up periods 1 and 2 months after the intervention (RPQ13 change (mean ± SD): at 1 month, Active = −21.8 ± 6.6, Sham = −2.2 ± 9.8; at 2 months, Active = −21.2 ± 5.3, Sham = −5.4 ± 13.7). No improvement was found in the subgroup with longer duration injuries. The results support rTMS as a tolerable and potentially effective treatment option for individuals with a recent (<1 year) concussion.

## Introduction

In most cases of mild traumatic brain injury (mTBI), also called concussion, the symptoms disappear in the first 2 to 4 weeks^1,2^. However, the symptoms can also persist for months or years following the injury; in that case, they are referred to as persistent post-concussion syndrome (PCS)^3,4^. Many authors consider symptoms lasting more than one month as PCS^2,5^, however, the more conservative DSM-IV guideline defines symptoms lasting more than 3 months as PCS^6^. The PCS symptoms include somatic symptoms (i.e., headache, blurry vision, anxiety, etc.) and cognitive (i.e., confusion, memory) deficits^1,7,8^. In 20–40% of mTBI cases symptoms are still reported at 6 months post-injury^9^, and in 10–20% of cases symptoms are still present at 1 year and beyond^10^. It should be noted that some of the symptoms reported in^9^ may have other causes besides mTBI.

Given that PCS imposes substantial medical and socio-economic burdens on patients and the healthcare system^11–13^, there is an urgent need to develop an effective treatment strategy as well as quantitative methods to monitor PCS recovery. The current treatments for PCS include medications^14^ and psychological treatments^15–17^. However, the effectiveness of these treatments is still in dispute^1^. In recent years a few studies have considered applying repetitive Transcranial Magnetic Stimulation (rTMS) as a treatment for PCS/mTBI^18–21^.

rTMS treatment involves the repetitive application of a quickly changing magnetic field pulse to the brain^22^. The rapidly changing magnetic field induces an electric field and causes ions to flow in the brain tissue. These current flows cause neurons in the area of effect to either depolarize or hyperpolarize. Depending on the frequency and site of pulse application, the effect can either increase or decrease cortical excitability for a period of time following the stimulation. It is generally thought that high frequency pulses (>5 Hz) will increase cortical excitability in a similar manner to Long-Term Potentiation^23,24^. rTMS is a non-invasive procedure and is usually well-tolerated however side effects such as seizures, the most serious TMS-related acute adverse effect, do occur but are extremely rare^25,26^. Other side effects like transient headache, local pain, toothache, and paresthesia are more common^25^. Many rTMS studies have been applied to treat Depression and while most had positive outcomes a number indicate there is no therapeutic effect^25,26^. More specifically, studies have shown some beneficial effects of rTMS treatment on patients with TBI^18,19,21^. A recent small (n = 15) open-label study of high-frequency rTMS applied to mTBI participants support the tolerability of rTMS treatment in a PCS population and show a significant improvement in PCS Symptom Scale score^18^. As PCS signs or symptoms can often last for a year, we believe that injured nerves and their connectivity can typically go through a recovery period which can last for one year suggestive that the application of rTMS within this recovery period is likely more beneficial than for periods longer than one year. In support, rTMS has been shown as an effective treatment of aphasia 6 months or more post stroke onset^27^. This suggests evaluation of rTMS in a controlled and randomized trial is warranted.

The dorso-lateral pre-frontal cortex (DLPFC) is a common site for application of rTMS. This site is near the surface of the brain and reachable using a typical figure-8 coil. The DLPFC is known for its involvement in the executive functions, which is an umbrella term for the management of cognitive processes^28^, including working memory, cognitive flexibility^29^, and planning^30^. The DLPFC has primary and secondary association areas including posterior temporal, parietal, and occipital areas, and is described in the pathophysiology of concussion^31^. In addition, the DLPFC has a significant role in acetylcholine and dopamine production and modulation; these neurotransmitters have significant role in restoring normal cognitive function^31^. There is evidence that stimulation of DLPFC area can be an effective treatment for depression^32^ and Alzheimer’s^33^ and PCS^18^.

In this randomized and double-blind pilot study, we evaluated rTMS treatment efficacy using the Rivermead Post Concussion Symptoms Questionnaire (RPQ)^34,35^ as the primary outcome measure. The secondary outcome measures were the Montgomery–Åsberg Depression Rating Scale (MADRS) for investigating the confounding effects of depression and a novel feature (the average field potential area, see methods) derived from Electrovestibulography (EVestG)^36^ recordings.

EVestG records vestibulo-acoustic predominantly vestibular^37,38^ neural activity. The EVestG technique^36^ provides a quantitative indirect measure of activity in various brain regions and neural pathways, particularly in the vestibular nucleus and vestibular peripheral apparatus. There is support for the use of EVestG features in PCS studies given: the prevalence of the dizziness or imbalance in the concussion population is 23–81%^39,40^; that vestibulopathy after mTBI^41^ can have a central axonal injury component and; the use of EVestG features in previous PCS studies have achieved a control versus PCS classification accuracy of 84%^42^ as well as been shown to be correlated with the Rivermead Post-Concussion Questionnaire (RPQ)^42,43^. The EVestG feature used for PCS is called the AP-area. It is the action potential (AP) area of the extracted FP of each ear’s signal (for details see Methods Section). This AP-area feature was postulated to be related to Na+ and/or Ca++ channelopathies and the buildup of plaques of amyloid β^42^. Thus, EVestG signals appear useful in characterizing change following a concussive impact.

In this paper, we present the results of the first placebo-controlled double-blind rTMS study as a treatment for patients with PCS. In addition, we investigated whether the duration of PCS has any impact on the response to rTMS; this has not been investigated previously. We hypothesize: 1) Active rTMS treatment improves the cognitive state of PCS participants significantly more than the Sham, 2) the EVestG feature specific to PCS, the AP-area, has a high correlation with the clinical symptoms of PCS as measured by RPQ, and 3) The improvement (if any) is more pronounced if TMS is received in less than one year after the head trauma.

To test our hypotheses, we compared the outcome measures, recorded at baseline, immediately post-treatment, and at two follow up times 1 and 2 months post-treatment (FU1 and FU2, respectively) with either Active or Sham rTMS treatment.

## Results

**Figure 1:**
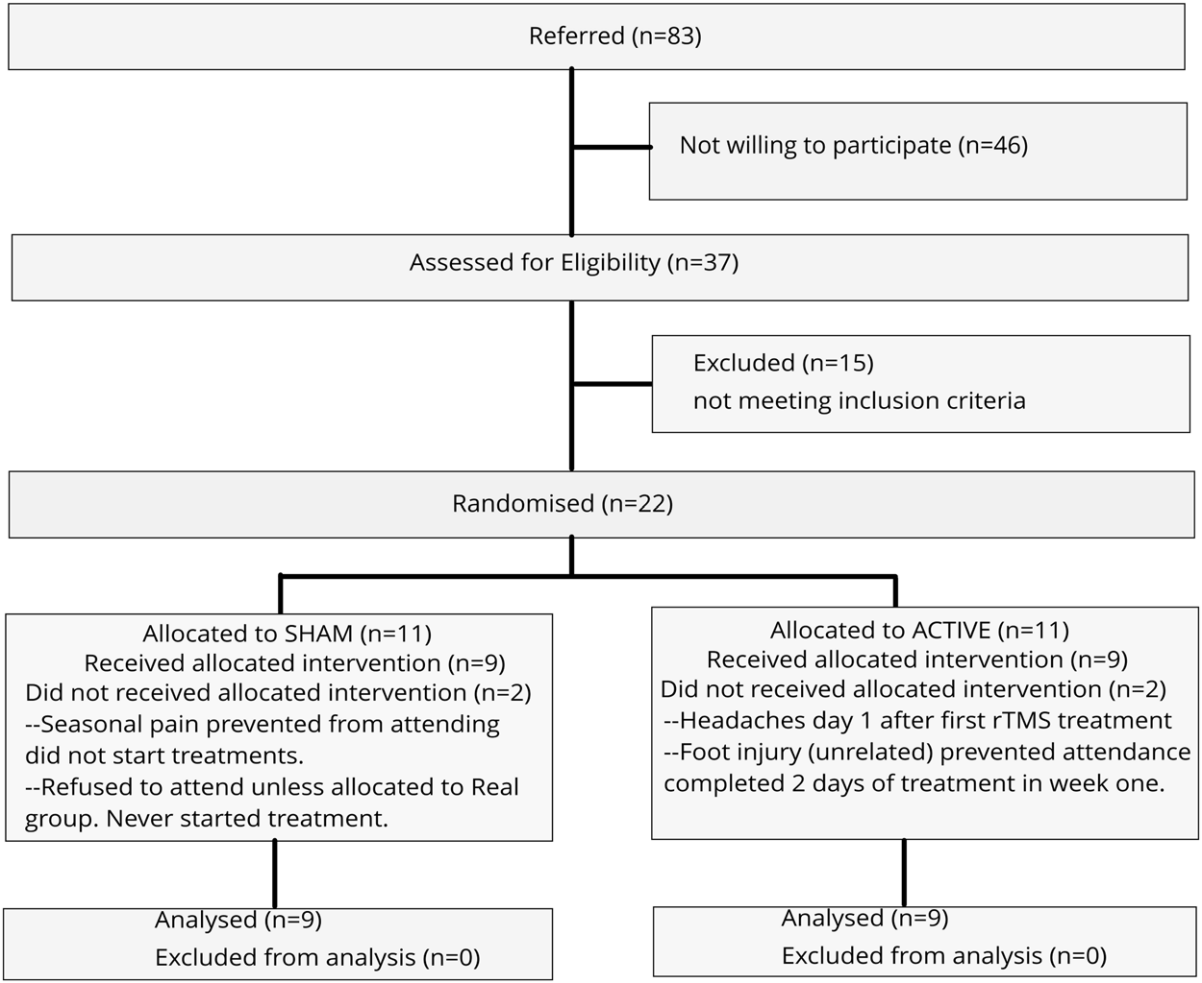
Flow chart of enrollment and treatment.

A total of 83 individuals with PCS were referred as candidates for this study, out of which 46 were not interested in participating and 15 did not meet all the inclusion criteria. The remaining 22 were enrolled into the study, of which 4 discontinued the treatment (3 for non-related reasons (travel) and one due to excessive headache due to rTMS treatment); data of those four were excluded from the analysis. Thus, data of 18 participants (9 males, age: 49.5 ± 12.4 (SD) years), who completed the treatment protocol, were analyzed. Figure 1 shows the details of recruitment and enrollment. Overall, the rTMS treatment was found tolerable by participants with PCS. Out of the 22 enrolled, 5 reported mild headache, while one participant discontinued due to excessive headache that worsened after the first day of treatment. No other side effects were reported. Of the 18 participants who completed the treatment 3 had one or two missing follow-up assessments. In those cases, the Last Observation Carried Forward method was used as an imputation statistical technique to handle the missing values in the analysis. No participant missed the baseline or immediate post-treatment assessments. Table 1 presents participant demographics and average outcome assessment scores resulting from the application of rTMS treatment to the left pre-frontal cortex (see methods for details). In terms of age, gender or duration past the injury comparative group averages were within one standard deviation (SD) of each other. There is no well accepted timeline for short (SPCS) versus long (LPCS) term PCS. However, based on neuropsychological assessments cognitive function mostly recovers within 1–3 months’ post-injury and some improvement can take place during the first year, however, some patients can remain impaired longer than one year^44,45^. Hence, the selection of 3 months and 1 year as time frames for SPCS and LPCS, respectively. The duration of injury in short-term PCS participants (SPCS) ranged from 4.5 to 11.5 months, while it was between 1.2 years to 4.8 years for the long-term PCS (LPCS) participants.

**Table 1:** Patient Demographics and average ± standard deviation (SD) outcome scores: Treatment was at a frequency of 20 Hz in trains of 30 pulses with an inter-train interval of 28.5 sec at 100% of resting motor threshold. 25 pulse trains were delivered (750 pulses/day) at each of 13 treatment sessions (10 in first 2 weeks and 3 in third week). base = baseline, post = post treatment, FU = follow up (FU1 = 30 days after treatment, FU2 = 60 days after treatment). MOCA was only applied at inclusion screening. In the MADRS baseline column superscript indicates a subject was on depression related medication(s) (^1^Lamictal, Zeldox, Zoloft, Clonazapam, ^2^Trazadone, ^3^Amitriptyline, ^4^Amitriptyline).

Groups	Age ± std	Year since injury	Sex	MOCA base	RPQ3				RPQ13				MADRS				EVestG AP-area			
Sham					base	post	FU-1	FU-2	base	post	FU-1	FU-2	base	post	FU-1	FU-2	base	post	FU-1	FU-2
LPCS, n = 5	56.8 ± 10.8	2.5 ± 1.2	3M, 2F	27.0 ± 3.9	6.6 ± 3.8	5.6 ± 2.6	6.2 ± 3.6	5.8 ± 3.5	28.5 ± 12.7	30.6 ± 7.8	27.1 ± 6.5	28.6 ± 9.3	9.2^1,4^ ± 6.6	6.8 ± 5.0	8.4 ± 5.7	7.8 ± 1.5	33.9 ± 11.3	33.0 ± 13.5	33.0 ± 11.3	36.5 ± 12
SPCS, n = 4	49.3 ± 5.4	0.7 ± 0.2	1M, 3F	27.3 ± 1.7	8.9 ± 3.4	6.8 ± 3.9	5.5 ± 2.8	4.3 ± 3.1	34.6 ± 5.7	28.4 ± 15.2	34.3 ± 14.4	29.3 ± 19.1	20.3 ^1^ ± 12.9	18.0 ± 15.9	12.3 ± 12.0	17.0 ± 12.7	20.0 ± 9.3	28.0 ± 10.8	27.2 ± 16.3	27.8 ± 11.9
All, n = 9	53.0 ± 8.8	1.6 ± 1.2	4M, 5F	27.1 ± 3.0	7.6 ± 3.6	6.1 ± 3.1	5.9 ± 3.1	5.1 ± 3.2	31.2 ± 10.2	29.6 ± 10.9	29.8 ± 9.9	28.9 ± 13.5	14.1 ± 10.8	11.8 ± 11.9	9.9 ± 8.0	11.9 ± 9.2	27.7 ± 12.2	30.8 ± 11.9	30.8 ± 12.6	32.2 ± 12.0
**Active**																				
LPCS, n = 5	50.8 ± 17.4	2.8 ± 1.3	4M, 1F	27.4 ± 0.9	5.7 ± 2.0	6.4 ± 2.9	5.8 ± 2.5	6.7 ± 2.0	24.5 ± 5.7	23.8 ± 9.6	23.6 ± 10.2	24.6 ± 12.3	12.0 ^2^ ± 5.2	11.6 ± 5.5	15.0 ± 8.9	15.6 ± 12.0	30.7 ± 7.1	29.5 ± 10.1	28.1 ± 11.8	33.5 ± 11.0
SPCS, n = 4	42.5 ± 15.4	0.8 ± 0.2	1M, 3F	29.0 ± 0.8	10.3 ± 1.7	7.0 ± 3.4	5.0 ± 4.1	3.8 ± 3.8	41.5 ± 6.9	33.4 ± 7.6	16.8 ± 11.0	17.5 ± 10.0	19.0 ± 5.7	12.3 ± 4.0	6.0 ± 2.0	5.0 ± 1.0	19.8 ± 2.1	34.0 ± 8.4	34.3 ± 9.0	41.8 ± 8.0
All, n = 9	46.7 ± 15.3	1.8 ± 1.4	5M, 4F	28.1 ± 1.2	7.7 ± 3.0	6.7 ± 2.9	5.5 ± 2.9	5.6 ± 2.9	32.1 ± 10.7	28.1 ± 9.6	21.1 ± 10.3	21.9 ±11.3	15.1 ± 6.3	11.9 ± 4.6	11.6 ± 8.3	11.6 ± 10.6	25.9 ± 7.8	31.5 ± 9.1	30.4 ± 10.6	37.2 ± 10.2

### Statistical Analysis

We analyzed the outcome measure data using a double multivariate Analysis of Repeated Measures ANOVA with “Time” as a within-subjects factor with four levels (baseline, post-treatment, follow-up 1 and follow-up 2), “Treatment Group” (Sham/Active) and “Time Since Injury” (SPCS/LPCS) as a Between-Subjects factors. In all instances, a p-value ≤ 0.05 was considered significant.

Table 2 show the significant effects and interactions. This is broken into Multivariate analysis, Mauchly test of sphericity, Within-subject effects, Univariate analysis and Pairwise comparisons. A lay description of these results impacts on outcome measures follows. Multivariate tests (Table 2) show there are no significant Main effects. However, the Between-Subjects interaction Sham_Active*LPCS_SPCS (*F(4, 11)* = *6.305, p* = *0.007, Wilks-λ* = *0.304, η*^2^ = *0.696*) and Within-Subjects interaction time*LPCS_SPCS (*F(1*2*, 3)* = *46.34, p* = *0.005, Wilks-λ* = *0.005, η*^2^ = *0.995*) were significant (SPSS V24). When the LPCS only population was considered there was a Simple effect for Sham/Active (*F(4*, *5)* = *6.635, p* = *0.031, Wilks-λ* = *0.159, η*^2^ = *0.841*).

**Table 2:** A summary table of significant effects and interactions from a repeated measures MANCOVA analysis performed using SPSS V24 using 4 repeated measures at times baseline, post-treatments and FU1 and FU2. Between subject groupings were Active_Sham and LPCS_SPCS. Dependent variables were RPQ3, RPQ13, MADRS and EVestG (AP-area). Tests in order are: Multivariate tests, Mauchly’s test of sphericity, Tests of Within-subject effects, Univariate tests, and Pairwise Comparisons.

Multivariate Tests		Wilkes-Lambda		Hypothesis	Error	Sig.	Partial eta	Observed
Effect (a)		Value	F	df	df		squared	power (c)
**Between all Subjects**	Sham_Active * LPCS_SPCS	0.304	6.305 (b)	4	11	0.007	0.696	0.918
Between LPCS Subjects	**Sham_Active**	0.159	6.635 (b)	4	5	0.031	0.841	0.746
**Within all Subjects**	time * LPCS_SPCS	0.005	46.343 (b)	12	3	0.005	0.995	0.999
a All = Design: Intercept + Sham_Active + LPCS_SPCS + Sham_Active * LPCS_SPCS Within Subjects Design: time								
b Exact statistic, c Computed using p = 0.05								
**Mauchly’s Test of Sphericity**								
**Within Subjects Effect (population) (a)**		**Mauchly’s W**	**Approx. Chi-Square**	**df**	**Sig**.	**Greenhouse -Geisser**	**Epsilon (d) Huynh-Feldt**	**Lower -bound**
**time (SPCS only)**	MADRS	0.055	13.663	5	0.02	0.031	0.841	0.746
**time (Sham only)**	MADRS	0.121	12.089	5	0.036	0.031	0.841	0.746
Tests the null hypothesis that the error covariance matrix of the orthonormalized transformed dependent variables is proportional to an identity matrix.								
d May be used to adjust the degrees of freedom for the averaged tests of significance. Corrected tests are displayed in the Within-Subjects Effects table.								
**Tests of Within-Subjects Effects**								
**Multivariate (a,e)**		**Wilkes-Lambda Value**	**F**	**Hypothesis df**	**Error df**	**Sig**.	**partial eta squared**	**observed power (f)**
**population**		0.378	3.833	12	103.476	0	0.277	0.993
all	**time**	0.151	3.476	12	39.978	0.001	0.468	0.962
SPCS only	**time**	0.204	3.285	12	47.915	0.002	0.411	0.961
Active only	**time**	0.395	3.63	12	103.476	0	0.266	0.99
all	time * LPCS_SPCS	0.227	3.002	12	47.915	0.003	0.39	0.941
Active only	time * LPCS_SPCS	0.378	3.833	12	103.476	0	0.277	0.993
e Tests are based on average variables, f Computed alpha = 0.05.								
**Univariate Tests, Sphericity tested**								
**Source (population)**	**Measure**	**Type III Sum of Squares**	**df**	**Mean Square**	**F**	**Sig**.	**partial eta squared**	**observed power (f)**
**time**	**RPQ3**	66.421	3	22.14	8.454	0	0.377	0.989
**time**	**RPQ13**	554.644	3	184.881	6.581	0.001	0.32	0.959
**time**	**EVestG**	741.369	3	247.123	8.894	0	0.388	0.992
**time (SPCS only)**	**RPQ3**	119.898	3	39.966	10.98	0	0.647	0.995
**time (SPCS only)**	**RPQ13**	883.586	3	294.529	6.404	0.004	0.516	0.924
**time (SPCS only)**	**MADRS (g)**	461.25	1.87	246.475	7.862	0.008	0.567	0.867
**time (SPCS only)**	**EVestG**	1034.201	3	344.734	9.491	0.001	0.613	0.988
**time (LPCS only)**	**RPQ3**	38.353	3	12.784	6.987	0.002	0.5	0.952
**time (Active only)**	**RPQ3**	28.754	3	9.585	2.812	0.064	0.287	0.59
**time (Active only)**	**RPQ13**	596.562	3	198.854	9.581	0	0.578	0.99
**time (Active only)**	**EVestG**	688.983	3	229.661	9.218	0	0.568	0.988
time * LPCS_SPCS	**RPQ3**	67.199	3	22.4	8.553	0	0.379	0.989
time * LPCS_SPCS	**RPQ13**	443.046	3	147.682	5.257	0.004	0.273	0.904
time * LPCS_SPCS	**MADRS**	351.006	3	117.002	4.855	0.005	0.258	0.878
time * LPCS_SPCS	**EVestG**	539.952	3	179.984	6.477	0.001	0.316	0.956
time * LPCS_SPCS (Active only)	**RPQ3**	48.254	3	16.085	4.72	0.011	0.403	0.831
time * LPCS_SPCS (Active only)	**RPQ13**	562.368	3	187.456	9.032	0	0.563	0.986
time * LPCS_SPCS (Active only)	**MADRS**	303.633	3	101.211	4.584	0.013	0.396	0.819
time * LPCS_SPCS (Active only)	**EVestG**	550.843	3	183.614	7.37	0.001	0.513	0.962
g Greenhouse Geisser test applied rather than Sphericity assumed								
**Pairwise Comparisons**								
		**p, baseline to post**	**p, baseline to FU1**	**p, baseline to FU2**				
**time**	**RPQ3**	0.008	0.001	0.001				
	**RPQ13**		0.004	0.010				
	**MADRS**		0.003					
	**EVestG**	0.006	0.026	0.001				

Mauchly’s test (Table 2) indicated that the assumption of sphericity had not been violated for any of the dependent variables with the exception of the outcome measure MADRS for the SPCS and Sham only populations Within-Subject time effects.

Table 2 shows the results of testing for Within-Subject effects. There were significant effects for 1) time (baseline, post, FU1, FU2) (population = all PCS, *F(1*2*, 103.48)* = *3.83, p* < *0.005, Wilks-λ* = *0.378, η*^2^ = *0*.2*77*), (population = SPCS only, *F(1*2*, 39.98)* = *3.48, p* = *0.001, Wilks-λ* = *0.151, η*^*2*^ = *0.468*), (population = Active only, *F(12, 47.92)* = *3.29, p* = *0.002, Wilks λ* = *0.204, η*^*2*^ = *0.411*) and 2) the interaction time*LPCS_SPCS (population = all PCS, *F(12, 103.48)* = *3.63, p* < *0.005, Wilks-λ* = *0.395, η*^*2*^ = *0.266*), (population = Active only, *F(12, 47.92)* = *3.00, p* = *0.003, Wilks-λ* = *0.227, η*^*2*^ = *0.390*). These were the only significant effects. No significant Between-Subjects effects were observed.

The univariate test results (Table 2) showed that the significant effect of time was held for all of the dependent variables except MADRS. This was true for the SPCS population alone but included MADRS. For the LPCS (RPQ3) and the Active (RPQ3, RPQ13 and EVestG) only populations there was also a significant time effect. The interaction time*LPCS_SPCS was significant on all of the dependent variables for the all PCS and Active only populations. This significant interaction was also seen for RPQ3 Sham. The interactions between time and treatment group (Active/Sham) were not significant for any of the dependent variables. Pairwise comparisons (Table 2) of each dependant variable change from baseline to each subsequent test time (post, FU1, FU2) depicts significance for one or more time steps as well as for all dependant variables at time FU1.

For comparison, we also applied a non-parametric approach, the repeated measures permutation test. Since we used a simple randomization design, covariates were not controlled for. The permutation test results were similar to the ANOVA results. For repeated permutation analysis across time (post-treatment, FU1, FU2) between Sham and Active SPCS treatment groups the, RPQ13 change (measurement minus baseline) (p = 0.017) and MADRS change (p = 0.013) were significantly different whilst the EVestG change (p = 0.063) was marginally significantly different. Using repeated measures permutation testing when the test variable was time rather than group (Sham/Active), for the SPCS subgroup over time, the RPQ3 change (measurement minus baseline) (p < 0.001), RPQ13 change (p = 0.005), MADRS change (p = 0.004) and EVestG AP-area change (p = 0.009) measures all changed significantly.

All the above test batteries and Table 3 (baseline to post, FU1, FU2 dependant variable change data) support the SPCS subgroup showing significant improvement for Active beyond Sham. This is elaborated for each outcome measure below.

**Table 3:** Outcome measure change from baseline to time X (post, FU1, FU2). Data are presented as effect size, (Active change µ + SD, Sham change µ ± SD), 95% precision. The estimated effect size was generated using Cohen’s d (negligible =< 0.2, medium effect is ~0.5, large > 0.8). The 95% precision is calculated using the 95% confidence interval/2.

Time X	RPQ3	RPQ13
Post	0.19, (−1.06 ± 3.07 −1.50 ± 1.32), 3.14	−0.24, (−4.00 ± 6.56 −1.61 ± 9.64), 10.04
FU1	0.10, (−1.81 ± 3.77 −2.13 ± 2.70), 3.37	−0.70, (−8.75 ± 12.11 −1.69 ± 8.65), 10.77
FU2	0.26, (−1.69 ± 4.61 −2.50 ± 2.52), 3.21	−0.55, (−7.88 ± 12.67 −2.33 ± 9.74), 10.36
	**RPQ3 LPCS**	**RPQ13 LPCS**
Post	0.82, (0.70 ± 2.68 −1.00 ± 1.22), 3.04	−0.49, (−0.70 ± 4.09 2.10 ± 6.97), 8.33
FU1	0.23, (0.10 ± 2.79 −0.40 ± 1.14), 3.11	0.07, (−0.90 ± 5.41 −1.40 ± 9.07), 10.89
FU2	0.73, (1.00 ± 3.41 −0.80 ± 0.84), 3.62	0.00, (0.10 ± 7.40 0.10 ± 6.26), 9.68
	**RPQ3 SPCS**	**RPQ13 SPCS**
Post	−0.65, (−3.25 ± 2.06 −2.13 ± 1.31), 2.99	−0.19, (−8.13 ± 7.42 −6.25 ± 11.47), 16.72
FU1	0.00, (−5.00 ± 3.12 −5.00 ± 1.73), 5.72	−2.35, (−21.83 ± 6.60 −2.16 ± 9.83), 18.98
FU2	−0.74, (−6.17 ± 1.76 −4.63 ± 2.29), 4.11	−1.42, (−21.17 ± 5.30 −5.38 ± 13.65), 21.77
	**MADRS**	**EVestG AP**-**area**
Post	−0.11, (−3.22 ± 4.87 −2.33 ± 7.42), 8.23	0.26, (5.64 ± 10.75 3.08 ± 7.01), 9.07
FU1	0.38, (−2.38 ± 8.75 −5.5 ± 8.05), 8.84	0.20, (3.62 ± 12.17 1.49 ± 8.50), 11.25
FU2	−0.02, (−2.38 ± 11.24 −2.22 ± 8.26), 8.51	0.48, (11.35 ± 12.60 6.02 ± 9.28), 11.57
	**MADRS LPCS**	**EVestG LPCS AP**-**area**
Post	0.36, ((−0.40 ± 3.71 −2.40 ± 6.99), 8.16	−0.07, (−1.25 ± 5.71 −0.87 ± 4.74), 7,65
FU1	0.70, (3.00 ± 5.22 −0.80 ± 5.40), 7.97	−0.21, (−2.69 ± 8.45 −0.88 ± 9.10), 12.81
FU2	0.61, (3.60 ± 9.15 −1.40 ± 7.13), 11.97	−0.24, (2.79 ± 7.31 4.24 ± 4.14), 9.76
	**MADRS SPCS**	**EVestG SPCS AP-area**
Post	−0.65, (−6.75 ± 3.86 −2.25 ± 9.03), 12.02	0.78, (14.26 ± 9.31 8.03 ± 6.50), 13.89
FU1	0.47, (−11.33 ± 3.79 −13.33 ± 4.62), 9.57	0.97, (14.13 ± 10.51 5.44 ± 7.04), 20.28
FU2	−1.01, (−12.33 ± 6.03 −3.25 ± 10.56), 17.72	1.27, (22.05 ± 8.78 7.81 ± 13.24), 19.44

### Rivermead Results

**Figure 2:**
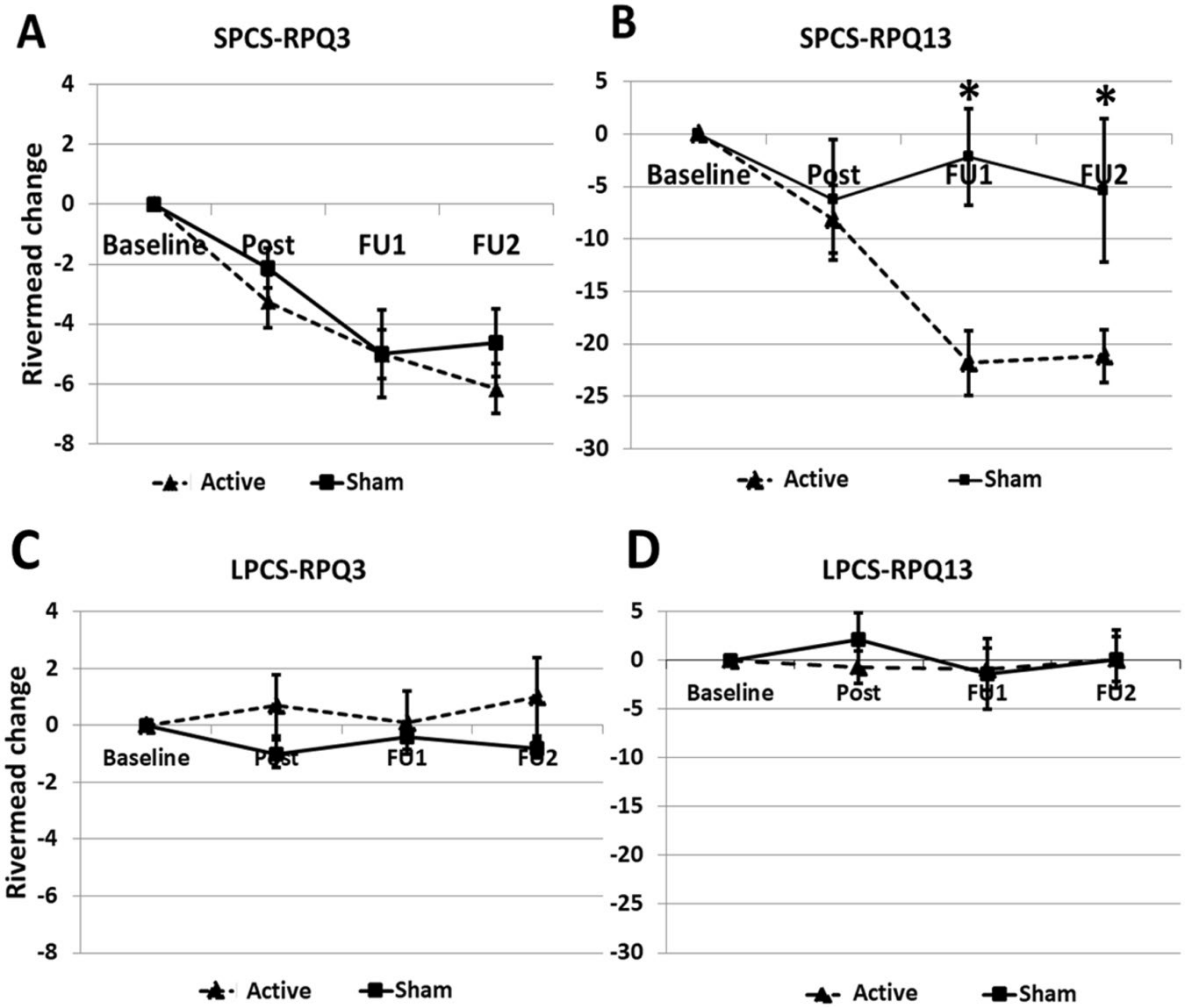
(**A**,**B**) Rivermead post-concussion questionnaire (RPQ) difference in each follow-up assessment from baseline. Each point is the average change ± standard error (SE) of all short-term PCS (SPCS) participants in each subgroup: Active (n = 4) and Sham (n = 4). (**A**) RPQ3 are the score of the first three symptoms in RPQ. (**B**) RPQ13 are the score of the last 13 symptoms in RPQ. (**C**,**D**) RPQ difference in each follow-up assessment from baseline. Each point is the average change ± SE of all long-term PCS (LPCS) participants in each subgroup: Active (n = 5) and Sham (n = 5). (**C**) RPQ3 are the score of the first three symptoms in RPQ. (**D**) RPQ13 are the score of the last 13 symptoms in RPQ. (*) indicate for significant difference.

Based on the above statistical analyses, for RPQ we found the following significant affects. The RPQ3 score for the SPCS subgroup decreased significantly for both Active and Sham during the follow-up assessments (Fig. 2A). We can see a slight change in the SPCS RPQ13 score immediately post-rTMS treatment for both Active and Sham subgroups, however, there was a significant large decrease (improvement) in this score at the first and second follow-up assessments for participants who received an Active but not Sham TMS treatment (Fig. 2B, Table 3). These improvements, however, were not observed for either Active or Sham treatment in the LPCS subgroup (Fig. 2C,D, Table 3).

### EVestG Results

**Figure 3:**
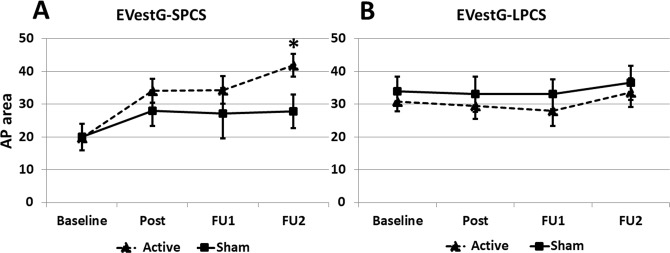
The average ± SE of the calculated feature (AP-area) extracted from the EVestG signal in all four assessments. (**A**) Short-term PCS participants who received Active (n = 4) and Sham (n = 4) treatments. (**B**) Long-term PCS participants who received Active (n = 5) and Sham (n = 5) treatments. (*) indicate for significant difference.

Overall, the EVestG and RPQ data showed similar trends (Fig. 3). Based on the above statistical analyses, for EVestG we found the following significant affects. For SPCS the EVestG feature, AP-area, increased (implying improvement) at the immediate post-treatment assessment for both sham and active treatment. During the follow-up assessments this improvement continued but only for Active group and the improvement difference between the Active and Sham intervention groups for SPCS subjects became significant at FU2 (Fig. 3A, Table 3). As in the Rivermead data, these improvements were not observed in the LPCS subjects’ data (Fig. 3B). In particular, the improvement from baseline to post-treatment was not observed for LPCS.

After considering all data (SPCS, LPCS, N = 68) from the 4 recording times (baseline, post, FU1, FU2) it was found AP-area is significantly (** = 0.01, * = 0.05) correlated with RPQ (RPQ3 plus RPQ13) but not MADRS (r = −0.36*, −0.53**, −0.28*, −0.02 respectively, Table 4, Pearson). More specifically, for the SPCS Active subgroup (N = 14) AP-area was observed significantly correlated with RPQ (RPQ3 plus RPQ13) as well as MADRS (r = −0.79**, −0.78**, −0.76**, −0.55*, respectively, Table 4). From the lowest parts of Table 4 which also look at the subgroups we can make the following observations: (1) There is a high correlation between AP-area and RPQ (RPQ3 plus RPQ13) for the Sham LPCS subgroup (r = −0.82**, −0.80**, −0.75**, respectively); (2) Data support rTMS affecting MADRS (thus the depressive components of RPQ13) for the Active LPCS subgroup and these two measures are correlated with AP-area (r = 0.48*, −0.47*, respectively) and; 3) Data indicate only a potential correlation between AP-area and RPQ3 (r = −0.53*) for the Sham SPCS group. As looking across all time may add variance to the data we considered baseline data (after verifying normality) only (SPCS, LPCS, N = 18) to find AP-area remained significantly correlated with RPQ (RPQ3 plus RPQ13) but not MADRS (r = −0.56*, −0.52**, −0.54*, −0.33, respectively).

**Table 4:** Table of measured correlations (Parametric–Pearson and Non-Parametric–Spearman-Rho) between the AP-area EVestG feature, RPQ (RPQ3 and RPQ13) and MADRS. All measures were verified as normally distributed based on their Kurtosis and Skew values. The upper rows refer to correlations for the all PCS (SPCS and LPCS) data considered across the four times (baseline, post, FU1, FU2). The lower 4 blocks refer to each of the Active intervention SPCS, Sham intervention SPCS, Active intervention LPCS and Sham intervention LPCS subgroup data each considered across the four times (baseline, post, FU1, FU2).

All PCS (N = 68), 2 tail test, * = 0.05 and ** = 0.01 significance				
	RPQ (correlation, p)	RPQ3 (correlation, p)	RPQ13 (correlation, p)	MADRS (correlation, p)
AP-area Pearson	−0.357** (0.003)	−0.529** (0.000)	−0.280* (0.021)	−0.022 (0.856)
AP-areaSpearman-Rho	−0.292* (0.016)	−0.449** (0.000)	−0.245* (0.044)	−0.043 (0.726)
Active SPCS subgroup (N = 14), 2 tail test, * = 0.05 and ** = 0.01 significance				
AP-area Pearson	−0.785** (0.001)	−0.780** (0.001)	−0.764** (0.001)	−0.547* (0.043)
AP-area Spearman-Rho	−0.719** (0.004)	−0.728** (0.003)	−0.697** (0.006)	−0.553* (0.040)
Sham SPCS subgroup (N = 15), 2 tail test, * = 0.05 and ** = 0.01 significance				
AP-area Pearson	−0.145 (0.605)	−0.526* (0.044)	−0.028 (0.920)	0.415 (0.124)
AP-area Spearman-Rho	0.071 (0.800)	−0.365 (0.181)	0.129 (0.647)	0.343 (0.211)
Active LPCS subgroup (N = 20), 2 tail test, * = 0.05 and ** = 0.01 significance				
AP-area Pearson	0.434 (0.056)	0.105 (0.661)	0.475* (0.034)	0.474* (0.035)
AP-area Spearman-Rho	0.512* (0.021)	0.265 (0.259)	0.504* (0.024)	−0.468* (0.037)
Sham LPCS subgroup (N = 19), 2 tail test, * = 0.05 and ** = 0.01 significance				
AP-area Pearson	−0.819** (<0.001)	−0.804** (<0.001)	−0.750** (<0.001)	−0.452 (0.052)
AP-area Spearman-Rho	−0.770** (<0.001)	−0.729** (<0.001)	−0.697** (0.001)	−0.486* (0.035)

Lastly, by using the change in outcome measure from baseline to FU2 following active treatment as an example, we observe there is a larger decrease in RPQ score (improvement) and increase in EVestG AP width (improvement) for those subjects with higher RPQ scores at baseline—this is typically the SPCS group of subjects (see Table 1). On average the largest improvement is seen in SPCS subjects which also have higher RPQ scores. More particularly, for the SPCS subjects receiving active treatment: 1) the correlations between RPQ or RPQ3 at baseline and the change in RPQ3 from baseline to FU2 were significant (r = 0.509* and r = 0.611*, respectively) and; 2) the correlations between AP-area at baseline and the change in RPQ and RPQ13 from baseline to FU2 were significant (r = −0.538* and r = 0.513*, respectively).

### MADRS Results

**Figure 4:**
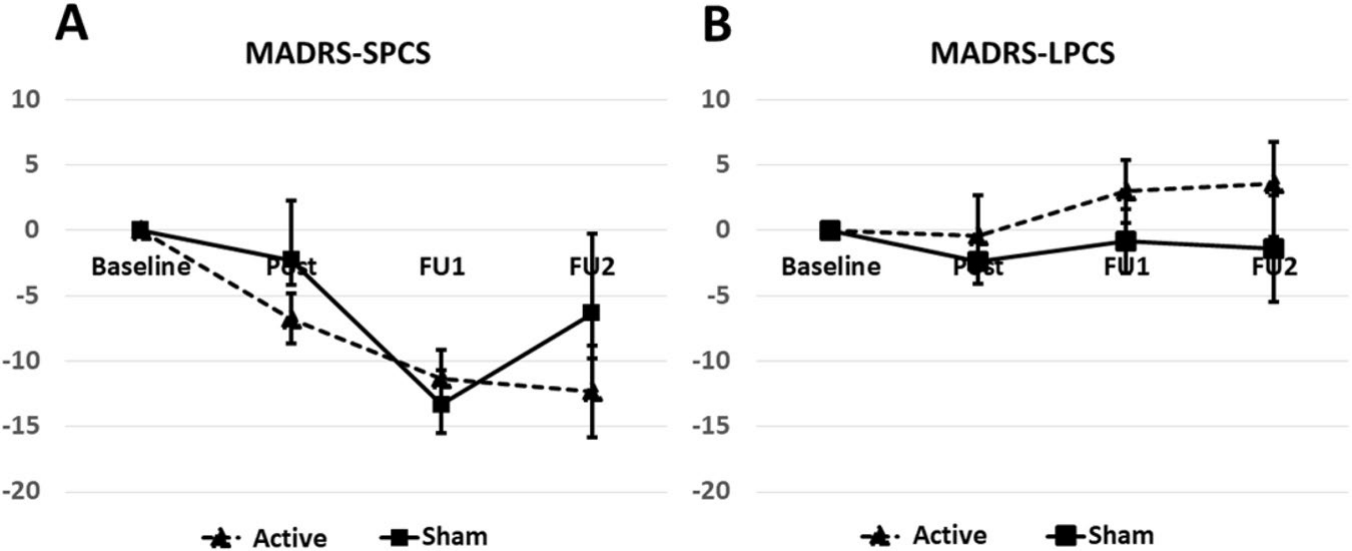
MADRS score difference in each of the follow-up assessments from the baseline. Each point is the average change ± SE of (**A**) Short-term PCS participants (SPCS) who received Active (n = 4) and Sham (n = 4) treatment. (**B**) Long-term PCS participants (LPCS) who received Active (n = 5) and Sham (n = 5) treatment.

The average MADRS score at baseline for the 18 participants was 14.1 ± 2.1 (SE); implying the majority of the participants had mild depression (two of the participants were moderately depressed). The average MADRS scores at baseline for SPCS and LPCS subgroups were: 19.6 ± 3.3 (SE) and 10.6 ± 1.8 (SE), respectively. The change in MADRS scores during the follow-up assessments compared to baseline for each of the Active and Sham subgroups of SPCS and LPCS participants are shown in Fig. 4.

Based on the above section’s statistical analyses, for MADRS we found the following significant affects. There was a significant improvement in MADRS scores of both Active and Sham SPCS individuals (Fig. 4A). Despite this there was a significant difference between the Active and Sham subgroups (Table 3). Perhaps the passage of time and or more so the rTMS treatment had a positive effect. On the other hand, the MADRS scores did not improve (or change) significantly for LPCS individuals in either Active and Sham subgroups. However, there was a significant difference between the LPCS Active and Sham subgroups (Table 3) that is in part linked to their quite different baseline values. Given the LPCS values are on average closer to control values there is less room for rTMS to improve symptoms thus the expectation is for more of a change in the SPCS group since their values were farther from baseline.

The data in Table 3 show the change from baseline to each time (post, FU1, FU2) for Active and Sham (bracketed middle entry in each cell). In this table the measure Effect Size (first entry in each cell) is presented to emphasise the size of the outcome measure differences without confounding this difference with sample size. Table 3 provides supporting evidence that despite N being small in sub-groups the observations that, in particular, the SPCS group receiving Active rather than Sham treatment showed the most improvement in RPQ13, EVestG and MADRS after rTMS and in particular in FU2.

### General Results

The independent t-test for equality of means with equal variances not assumed was applied. There was no significant difference between SPCS or LPCS subgroup (sham versus active) baseline RPQ3 (LPCS, p = 0.65; SPCS, p = 0.50), RPQ13 (p = 0.54, p = 0.17), MADRS (p = 0.48, p = 0.87) or AP-area (p = 0.61, p = 0.97) values. Compared to SPCS baseline values the average LPCS baseline values for RPQ3 (p = 0.02), RPQ13 (p = 0.01) and MADRS (p = 0.03) were smaller, whilst the average AP-area (p < 0.01) value was larger. These all correspond to decreased symptom severity. This is anticipated given there is a “separate” recovery period associated with the SPCS timeframe. Given that the SPCS population had more females and LPCS more males it is reasonable to ask if this difference is gender based. The study in [54] suggests not. There was no significant difference between male and female sham or active subgroup baseline RPQ3 (LPCS, p = 0.23; SPCS, p = 0.95), RPQ13 (p = 0.11, p = 0.66), MADRS (p = 0.59, p = 0.42) or AP-area (p = 0.59, p = 0.28) values. When we divided the population into the 9 oldest and 9 youngest and compared baseline RPQ, RPQ3, RPQ13, AP-area, MOCA and MADRS values, there were no statistically meaningful differences (p = 0.34, 0.52, 0.32, 0.63, 0.12, 0.81, respectively).

Lastly, although the information regarding patients’ Glasgow Coma Scale at the time of head trauma was not available, three patients indicated that they had loss of consciousness after head trauma and they all belonged to SPCS group. Therefore, in our study, severity of the symptoms at the time of impact could not be correlated with the length of recovery.

## Discussion

A primary outcome measure was the RPQ13 score; it showed a significant improvement for rTMS Active compared to Sham treatment for the SPCS subgroup but not for the LPCS subgroup (Fig. 2). At the post-treatment assessment, the improvement of the SPCS individuals in the Active treatment group was only marginally better than those in Sham treatment group but by the FU1 and FU2 assessments the difference was significant (Table 3). This improvement persisted at least two months post-treatment. Based on this delayed and long-lasting response we speculate that rTMS may be modifying the pace of recovery rather than affecting the symptoms directly. Our observed delayed response is congruent with the fundamental rationale for therapeutic use of rTMS, which is based on compelling evidence that rTMS is able to modulate long-term neural plasticity at the network level^46–48^. These lasting neuroplastic changes induced by rTMS may help promote the recovery of brain function and decrease the burden of the disabling sequelae of a brain injury. The observed improvements measured by RPQ13 in the Active intervention are indicative that rTMS may indeed help recovery of the cognitive and or sensory impairments in individuals if applied within a few months past the injury.

RPQ3 was also a primary outcome measure. For either SPCS or LPCS subgroups, it showed no significant improvement for rTMS Active over Sham treatment (Fig. 2). The fact that the RPQ3 measurement improved substantially for both the Active and Sham SPCS but not either of the LPCS subgroups, suggests that the improvements in nausea, dizziness and headaches (RPQ3 measures) were not mostly due to rTMS treatment. It is likely that these participants (SPCS subgroup) were still experiencing the natural process of recovery from their injury and during this time an improvement in these symptoms would be expected^10,49^. This does not discount the possibility rTMS could lead to an improvement in RPQ3 scores^50^.

The feature extracted from EVestG measurements, the AP-area, showed a large improvement for SPCS but not for the LPCS subgroups; this SPCS improvement was significantly larger for Active relative to Sham treatment. These trends matched well with the RPQ13 results. However, there was also a smaller consistent improvement in AP-area from post-treatment to FU2 for the Sham SPCS population. For RPQ3 there was also an improvement for Sham and Active SPCS which above was attributed to a “natural” recovery over time. Supporting the natural recovery hypothesis in^42^ it was shown, using the same AP-area feature used herein, that the SPCS cluster distribution was more distant (has a smaller AP-area feature) from the control cluster (has a large AP feature). The LPCS cluster lay between the two i.e. there was a “natural” recovery over time. “Dizziness”, as in RPQ3, appears likely to be a component in AP-area feature particularly in SPCS. This is supported by the Table 4 correlation data which show after considering all PCS data across the 4 recording times the AP-area feature is significantly correlated with RPQ (RPQ3 and RPQ13) but not MADRS. More specifically, the SPCS Active subgroup AP-area feature was observed significantly correlated with RPQ (RPQ3 and RPQ13) and MADRS. That is in SPCS depression symptoms were improved.

Since rTMS has been shown to be an effective treatment for depression^32^, there is a concern that improvements in a participant’s depression during treatment could confound the measurement of PCS-specific symptoms. To gauge the extent to which this may have occurred, the MADRS score was also measured at each assessment. In addition, EVestG analysis can also be sensitive to depression^51^ but, the EVestG feature used to diagnose depression was not the AP-area. Indeed, the AP-area feature in^38,51^ did not vary significantly between controls and a population with Major Depressive or Bipolar Disorder, and if there was any change it was to increase rather than decrease the AP-area. The results support previous evidence that AP-area may be an effective indicator of PCS symptom presence and or severity^42^.

As shown in Fig. 4A the depression level in both active and Sham subgroups of SPCS individuals improved with significantly larger improvements for Active compared to Sham treatment. Counter-intuitively, as shown in Fig. 4B, the depression level in the active (but not the Sham) subgroup of LPCS individuals appeared to worsen. An in-depth analysis of the MADRS data indicates that one participant from each of FU1 and FU2 had a particularly “bad” day relative to their other three MADRS measures; this may be in part responsible for this aberration.

One may question whether the observed improvement in the SPCS outcome measures is due to improvement of depression and/or PCS. Both are equally valid explanations of the results. EVestG depression studies^51,52^ showed no large variation in AP width (the key EVestG feature herein). This suggests the improvement may be in both depression and mTBI symptomatology. This is being explored further in a current study.

Most of the PCS participants (N = 15) had only one impact but two had two impacts and one had 3 impacts. No significant correlation was found between baseline symptoms’ severity and the number of impacts. This is expected as it is not necessary that the number of impacts positively correlate with PCS severity. It may be that someone who had one severe impact is being compared to someone with two light impacts. Moreover, the site of the impact is important^53–55^. Usually individuals with side/lateral impact are more likely to have severe symptoms compared to the ones with front or back impacts due to the head anatomy^55,56^.

We acknowledge the small size of the study as the main limitation of this study. As reported in the Method section, out of 83 referred patients approximately half were not interested or reluctant to receive rTMS treatment; the reason, in part, was due to the difficulty in attending three weeks of treatment sessions almost every day and in part, in our opinion, a reluctance to “risk” the new rTMS treatment. Also, many concussed individuals often have headache as one of their symptoms and they may pre-conceive themselves to be less tolerant to receive rTMS pulses to the head. However, there is recent evidence that rTMS can alleviate mTBI headache symptoms^50^. Given the small size of the data and 4 subgroups, we caution the interpretation of the statistical analysis of our results as the power of the test is low. Nevertheless, our results of tolerability and rTMS treatment efficacy are congruent to those of a similar study in small samples^18^, and encourage investigation of rTMS as a treatment for persistent PCS within a larger clinical trial. Other limitations include the visibly different Sham coil design and its sensory “poke” effect difference although a person who has not received rTMS before, would not be able to detect the difference. Anti-depression medication is also a potential limitation in four PCS subjects (Table 1). However, in^51^ and/or our own continuing studies we show the AP-area feature is largely independent of anti-depressant medication and the MADRS score. Lastly, the baseline MADRS depression levels between LPCS and SPCS were not matched meaning MADRS improvement may be potentially (and as observed) larger for the SPCS group different.

Future studies should determine the response rate of rTMS treatment on a medication-free PCS population, and whether the EVestG assessment is predictive for an individual’s response to rTMS. As the EVestG measure was significantly correlated with RPQ (p < 0.05), RPQ3 (p < 0.01) and RPQ13 (p < 0.05), it may prove useful as an assistive tool in monitoring recovery. The substantial improvement in the group with recent injuries as compared to the lack of improvement in the group with older injuries uniquely implies that the efficacy of the treatment is likely improved by administering it as soon as it is safely possible after the injury. If these results can be validated with a larger clinical trial, this tolerable treatment could bring great benefit to a group of people who suffer from the debilitating symptoms of PCS and currently have limited options to speed their recovery.

## Methods

### Study Design

The research design was a randomized, placebo-controlled and double-blind clinical trial. The participants, assessors of the baseline assessment and outcome measures and those who analyzed the EVestG data to extract the features, were blind to the group assignment. Participants were randomly (coin toss) assigned to one of two groups of Active or Sham rTMS treatment by the study coordinator. This study was approved by the University of Manitoba Biomedical Research Ethics Board, and all participants signed an informed consent form prior to participating in the study. All experimental procedures were performed in accordance with the protocol approved by the Biomedical Research Ethics Board and its regulations. Patients were primarily assessed at the Adult Medical Clinic, Victoria General Hospital and Traumatic Brain Injury Clinic, Winnipeg and referred by coauthors BM and JS. The rTMS intervention and all other assessments were conducted at the Riverview Health Center, Winnipeg, Manitoba.

### Intervention

A MAGSTIM alpha flat figure of eight coil (PN 3190) was used for Active and Sham stimulation of the left DLPFC (The MAGSTIM Company Ltd, Whitland Industrial Estate, Spring Gardens, Whitland Carmarthenshire. SA34 0HR, UK.). The MAGSTIM system was a Rapid 2 system incorporating BRAINSIGHT 2 navigation. To create the Sham coil a 19 mm thick piece of head-stage shaped cedar wood was slipped underneath the coil between the patient and coil to attenuate the strength of the induced electrical field in the brain tissue to a level well below the threshold. Simulations were performed in COMSOL MULTIPHYSICS (COMSOL, Inc., 100 District Avenue, Burlington, MA 01803, USA.) to estimate the reduction in field strength resulting from the insertion of the wooden block. Detailed coil specifications were provided by MAGSTIM which were used for this analysis.

A simulation of coil field strength was performed in COMSOL MULTIPHYSICS using a model of the coil based on dimensions provided by the manufacturer. The coil was positioned over a model of the subject’s head and brain created from two concentric spheres of radii 8.75 cm (head) and 8.00 cm (brain). The skull had a thickness of 0.75 cm and was given an electrical conductivity of 0.01 S/m, while the brain had a conductivity of 0.33 S/m. The wooden block was assumed to have a conductivity similar to air, which was modelled as 0.0001 S/m (non-zero value used to aid result convergence). It was found that by increasing the distance by 19 mm (the width of the wooden block used in the sham condition), the effective electric field strength as measured 1 mm below the surface of the brain was only 61.1% of the field strength without the block.

This means that the Sham condition would effectively be stimulating the tissue at 61.1% of resting motor threshold (RMT), well below the threshold where a significant number of neurons would be activated. This meant the coils were visually different but participants were generally oblivious to the head-stage changes between Sham and Active treatment as operator stood behind them holding the head-stage and slipping in the wooden barrier. Feel-wise the Sham had less of a “poke” sensation (based on the authors own sensations) meaning sensation could potentially be perceived as different. The sound produced was the same for Sham and Active head stages.

Table 5 shows the rTMS stimulus parameters applied in an example group of studies (most with small sample size) focusing on mTBI/TBI or Alzheimer’s Disease but not depression. Based on these example studies^18–21,33,57–59^ the stimulus frequency applied ranges from 10–20 Hz, resting motor threshold (RMT) is 80–110%, train duration is 1–5 sec, the number of trains is 10–25, all studies have the number of pulses less than the daily safety limit, stimulus times are most common over 2–4 weeks and the stimulus application site of the left dorsolateral prefrontal cortex (DLPFC) is most common. The parameters chosen for this study lie within those ranges and in particular match our earlier efficacious AD study^33^ but with the stimuli limited to 3 weeks and, as common with PCS/mTBI studies, only applied to the Left DLPFC (L-DLPFC).

**Table 5:** Table of example previous PCS/TBI and AD studies showing rTMS stimulus parameters. RMT = resting motor threshold. LDLPFC = left dorsolateral prefrontal cortex.

	Hz	% RMT	Train duration	# of trains	# pulses	Pathology, # of sessions, site
Koski, 2015^18^, N = 15	10	110	5 sec	20	1000	PCS, 20 sessions, LDLPFC
Cavinato, 2012^19^, N = 1	20	90	1 sec	10	200	TBI, 10 sessions, LDLPFC
Leung, 2016^21^, N = 24	10	80	1 sec	20	2000	Headache, Left motor cortex
Xia 2017^20^, N = 16	10	90	10 sec	10	1000	Vegetative Consciousness, 20, LDLPFC
Rutherford, 2015^33^, N = 10	20	100	1.5 sec	25	750	AD, 13, L&R-DLPFC
Bentwich, 2010^57^, N = 20	10	90-110	2 sec	20	1200	AD, 54, multiple sites
Devi, 2014^58^, N = 12	10/15	90	5 sec	20	1500	AD, 4, L&R-DLPFC
Rabey, 2013^59^, N = 15	10	90-110	2 sec	20-25	400-500/site	AD, 54 sessions, multiple sites

The pulses were given at a frequency of 20 Hz in trains of 30 pulses (1.5-second duration) with an intertrain interval of 10 seconds at 100% of the resting motor threshold. A total of 25 trains of pulses were given at each treatment session (750 pulses/day), which is well within recommended safety limits. Each participant received 13 treatment sessions were given to each participant over the course of three weeks: 10 sessions over the first two weeks and 3 sessions on the third week (Mon/Wed/Fri). The same protocol was used for Sham treatment. The study participants were assessed at baseline, immediately after the treatment block and in two more follow up assessments at days 30 and 60 after rTMS treatment (FU1 and FU2, respectively). Before commencement of any interventions we changed the originally proposed bilateral application of rTMS to only a left side application for 1) simplicity and compatibility with similar previous studies^18^, and 2) to limit any potential to aggravate depression levels as the majority of depression protocols apply only low frequency rTMS to the right side^60^.

The inclusion criteria were: 1) 19 years of age or older, 2) history of at least one or more head trauma with or without loss of consciousness in the last 5 years, 3) a Glasgow Coma Scale (GCS) > 13 within 10 minutes after the head trauma, 4) having continued symptoms (e.g. blurred/double vision, vertigo, headache, imbalance, mood/cognitive/sleep abnormalities) and signs (e.g. abnormal convergence insufficiency, eye misalignment, cerebellar/vestibular abnormality, cognitive abnormality) of PCS at least one month after the head trauma at the time of enrollment, and 5) normal hearing as determined by a hearing threshold screening test.

Most (N = 15) of the PCS participants had only one impact but a couple of them (N = 2) had two impacts and one had 3 impacts. The head injuries of participants included falls, sport and car accidents.

Participants were excluded if they had any other brain lesions, severe or recent heart disease, alcoholism, pregnancy, a history of epilepsy or seizures, metallic objects or pacemakers in the body (with the exception of dental implants), an inability to communicate in English, were active users of illicit drugs, or users of neuro- or psycho-active medications.

The trial was registered at ClinicalTrials.gov, number NCT02426749. Due to difficulty in recruiting and small sample size, we enrolled as we recruited. Therefore, allocation to Sham or Active groups was based on a coin flip.

### Outcome Measures

#### Rivermead Post Concussion Symptoms Questionnaire (RPQ)

The RPQ is a self-reported and reliable measure of PCS. It was originally developed as a measure of the severity of the symptoms post injury^34,35^. Scores from the 16 RPQ questions can range from 0 to 64 as symptoms are rated on a 4-point Likert scale, ranging from “not experienced at all” to “a severe problem”^61^. The scale is unbiased for age and gender^62^. Because a score of 1 for any item can indicate “no worse than before the injury” and if included in the total score, a cut-off value cannot be utilized for this measure. Providing mean scores for non-clinical (5.8^63^) and clinical populations (36.3^64^) can be somewhat arbitrary as is providing symptomatic ranging such as, 0–12 (minimally affected), 13–24 (mild), 25–32 (moderate), and 33 (severe) or more respectively^34^. The 16 questions making up RPQ should not be combined into single score but broken into RPQ-3 (headaches, dizziness and nausea) and RPQ-13 (cognitive and emotional) to form a unidimensional construct^35^. The RPQ-13 and RPQ-3 scales showed test-retest reliability coefficients of 0.89 and 0.72 (both p-values < 0.01)^35^. RPQ-3 and are commonly the ea3ly (immediate post-injury) symptoms associated with the PCS^35,65^, whilst RPQ-13 and are commonly the late symptoms associated with PCS^35,65^.

RPQ does have limitations. For example, PCS has measured in two group of participants; mTBI and non-head injury control (orthopedic control) using RPQ. The outcome showed patients with mTBI reported more post-concussion symptoms and fatigue than the controls at the beginning of recovery, but by 6 months after injury, did not differ as a group from non-head injury trauma controls on cognition, fatigue, or mental health^66^.

#### Electrovestibulography (EVestG)

**Figure 5:**
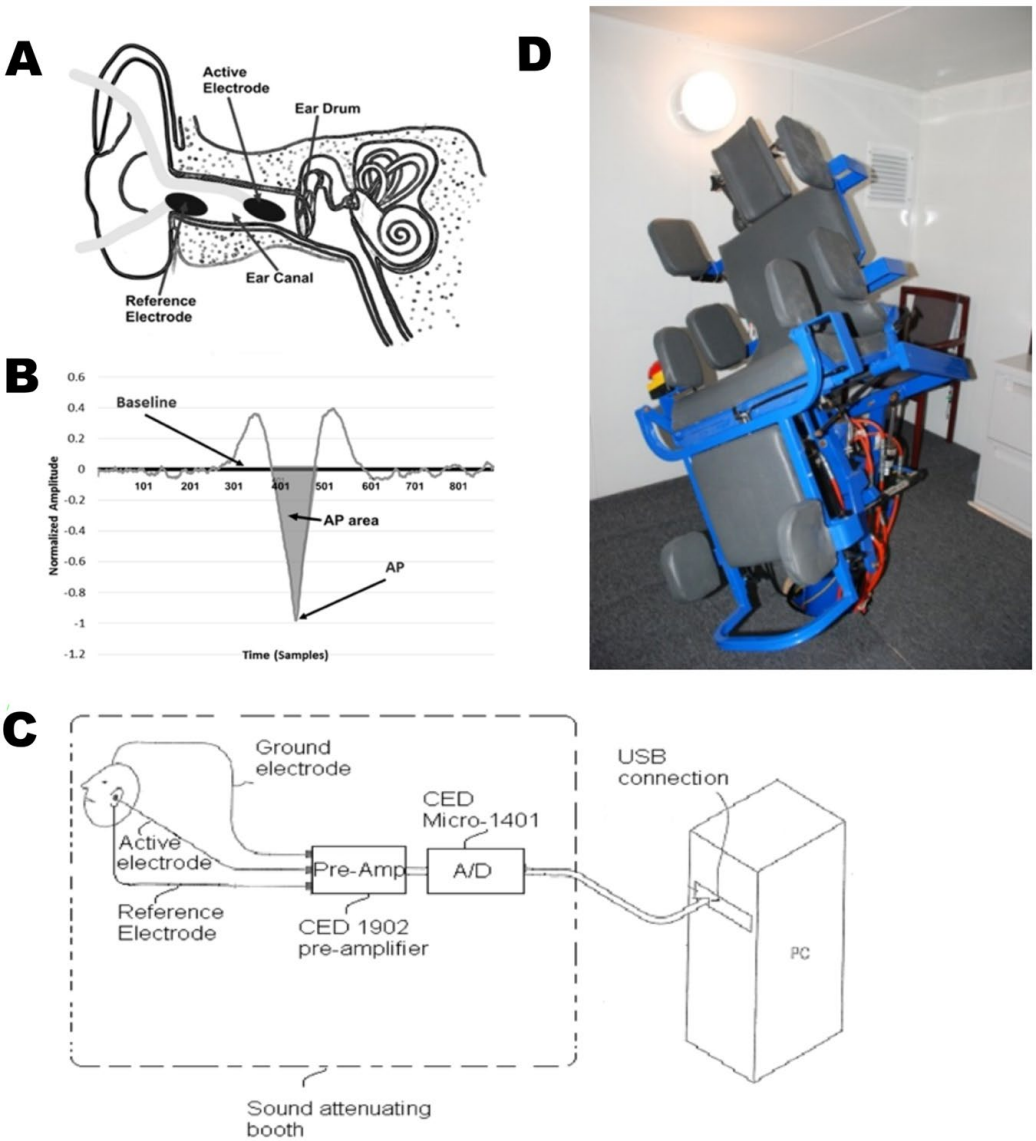
(**A**) Electrode connections and placement. (**B**) A typical normalized FP. The bounded area between the baseline and the AP point (marked area) is our calculated feature as the AP-area. (Horizontal scale 41.6 samples = 1 ms, Vertical axis in mV). (**C**) Recording configuration. (**D**) Hydraulic Chair inside anechoic room.

EVestG signals were recorded by placing electrodes inside of the ear canals close to the tympanic membrane (Fig. 5A), reference electrodes on each ipsilateral ear lobe, and a ground electrode (BIOPAC EL258S) on the forehead. The ear electrodes were silastic wrapped silver wire with the tip covered in cotton wool soaked in a mixture of saline and conductive gel to reduce interface impedance. Once the electrodes were placed, the participant sat on a hydraulic chair inside an acoustically attenuated and electromagnetically shielded chamber, and their static (background – no motion) and dynamic (passive whole-body movement) vestibulo-acoustic neural activity were recorded. In this pilot study, we only analyzed the signals recorded during static phase. The participants were instructed to close their eyes during the recording. The signals of both ears were recorded using a CED1902 Biological Amplifier, CED1401 Analogue to digital converter (ADC) (Fig. 5C) and using SPIKE2 (SPIKE2 Cambridge Electronic Design Limited Technical Centre, 139 Cambridge Road Milton Cambridge, CB24 6AZ, England) with a sampling rate of 41,666 Hz, and a 300 Hz high-pass filter to particularly remove muscle artifacts. The wavelet-based signal processing technique, called the Neural Event Extraction Routine (NEER)^36^, was used to detect spontaneous vestibular field potentials (FPs) buried in the noise. NEER utilizes a modified complex Morlet wavelet analysis of phase change across multiple scales and a template matching (matched filter) methodology to detect FPs buried in noise and biological and environmental artefacts^36^. Figure 5B is an example of the average detected FPs extracted by the NEER algorithm.

A recent pilot study^42^ has demonstrated that evaluation of vestibulo-acoustic performance using EVestG holds promise for identifying the presence of PCS. Similar to that study we calculated as the EVestG measure of PCS^42^ the action potential (AP) area (shaded region of Fig. 5B) of the extracted FP^42^ of each ear’s signal as the characteristic features to investigate the effect of rTMS treatment in this study. The AP-area is defined as the area between the horizontal zero axis and the AP point (shaded region of Fig. 5B). Note: The AP value (the peak of the downward curve) is normalised to −1mV to allow for electrode impedance effects, and the AP-area is mV-samples.

The feature used herein, was the average area under the normalized AP point (Fig. 5B) that is called AP-area. In a study recent pilot study^42^ the 95% confidence difference range for the AP-area was [41.7 to 46.3] for controls and [28.7 to 35.2] for PCS ([16.1 to 26.1] for SPCS and [31.8 to 38.0] for LPCS). Meaningful differences are selected herein to be greater than the 95% range differences. Clinically important ranges have not been validated yet. Good reliability for PCS measurement using AP-area has been obtained using an additional population of 21 PCS (7 SPCS and 14 LPCS) were classified using the same AP-area feature used in^42^; the resultant unbiased accuracy was 95%^43^. The increase in accuracy was a consequence of having a larger ratio of SPCS participants. This feature has been related to known channelopathies^42^. EVestG repeatability studies show there is a small diurnal variation^67^ but elsewise re-recordings across days at the same time are repeatable if the same SNR conditions are also repeated. All recordings were scheduled between 9am and 4 pm and where possible between 9am and 12 noon.

The recording Signal to Noise Ratio (SNR) was strongly dependant on the electrode impedance. To ensure the SNR was adequate for recording the recording background noise level (and inferred electrode impedance) was determined by examination of its power spectrum and comparison of its high and low frequency power bands.

In previous EVestG studies^42,43^ that showed promise for identifying the presence of PCS only the static phases were analyzed. Since we are investigating whether EVestG has the potential to monitor recovery as well, we used the same static background features. The analyses herein are made on background (BGi) segments recorded with the subject sitting upright and not being moved i.e. static segments, so motion artefact was normally not a serious issue. Following visual examination, segments that were markedly impacted by artefactual noise were omitted. In a full EVestG recording procedure there are normally 5 BGi segments recorded in the sitting position. In the sitting position, 5 BGi segments were generated in the 1.5 sec immediately prior to yaw (right), pitch (backwards), and roll (left and right) tilts as well as a vertical (upward) translation. In this study, only the static (no motion) BGi segments were analyzed. The responses to yaw, roll, pitch and vertical translation stimuli are data for use in a future study. Thus, one may consider the number of ‘repetitions’ (read BGi segments recorded) as 5, of which 3 BGi segments (prior to the roll left, roll right and pitch motions) were averaged and used for each subject. If any of those 3 BGi segments were artefactual, the up or yaw BGi segments replaced them. This segment selection procedure was used to match previous studies^42,43^.

The EVestG chair (Fig. 5D) including chair controller was designed by author BL and manufactured by NEURAL DIAGNOSTICS Pty Ltd. 16/537 Malvern Rd Toorak, Victoria 3142 Australia. The NEER^36^ software was designed by author BL and a license is available from NEURAL DIAGNOSTICS Pty Ltd. 16/537 Malvern Rd Toorak, Victoria 3142 Australia.

#### Montgomery–Åsberg Depression Rating Scale (MADRS)

MADRS is one of the most common used instruments in depression research^68^. It is a ten-item diagnostic questionnaire with a total score of 60 that is used to measure the severity of depression. Usually a score >6 indicates depression with 7–19 being considered asymptomatic-mild and >18 moderate-severe. Inter-rater reliability ranges from 0.89 to 0.97^68^. The internal consistency of the MADRS is considered very high, given the high correlation between all items (r = 0.95)^69^. Correlation of MADRS has been shown to be generally high or very high with other measures such as HAM-D (between 0.80 and 0.90)^70,71^, RDC (0.70)^72^, and with IDS-C (0.81)^72^. Since this study’s rTMS protocol is similar to that used clinically as a treatment for depression, we used MADRS to investigate whether there was any confounding effect from the treatment of depression symptoms.

### Data Analysis

Our study hypotheses were: 1) The Active rTMS treatment will improve the cognitive state of PCS participants significantly more than a Sham, 2) The PCS features of EVestG have a high correlation with the clinical symptoms of PCS measured by RPQ, and 3) The improvement (if any) is more pronounced if rTMS is received in less than one year after the head trauma.

To test these hypotheses, we used 1) a double multivariate Analysis of Repeated Measures ANOVA with “Time” as a within-subjects factor with four levels (baseline, post-treatment, follow-up 1 and follow-up 2), “Treatment Group” (active and Sham) as a between-subjects factor, and “Time Since Injury” (SPCS and LPCS) as a second between-subjects factor and 2) a post-hoc analysis. In all instances, a p-value ≤ 0.05 was considered significant.

Given the small sample study size (which means verification of the distribution may be unreliable) an alternate non-parametric approach is also indicated. Accordingly, a repeated measures permutation test (using the R software) was also be applied. (The R software is free and can be can be found/downloaded at: R Core Team (2016). R: A language and environment for statistical computing. R Foundation for Statistical Computing, Vienna, Austria).
